# 4D Flow Yields Similar Clinical Results Compared To 2D Phase Contrast for Decision Making Regarding Pulmonary Valve Replacement in Repaired Tetralogy of Fallot

**DOI:** 10.1007/s00246-025-04146-3

**Published:** 2026-01-06

**Authors:** Alison Almgren-Bell, Andrada Popescu, Aparna Sodhi, Michael Markl, Cynthia K. Rigsby, Joshua D. Robinson

**Affiliations:** 1 Northwestern University Feinberg School of Medicine, Chicago, IL USA; 2 Department of Medical Imaging, Ann & Robert H. Lurie Children’s Hospital of Chicago, 225 East Chicago Avenue #9, Chicago, IL 60611 USA; 3 Department of Radiology, Feinberg School of Medicine, Northwestern University, Chicago, IL USA; 4 Department of Biomedical Engineering, McCormick School of Engineering, Evanston, IL USA; 5 Division of Cardiology, Ann & Robert H. Lurie Children’s Hospital of Chicago, 225 E Chicago Ave, Box 21, Chicago, IL 60611 USA; 6 Department of Pediatrics, Feinberg School of Medicine, Northwestern University, Chicago, IL USA

**Keywords:** Cardiac MRI, 4D flow, Tetralogy of fallot, Pulmonary valve replacement

## Abstract

2D phase contrast (PC) CMR is critical for monitoring pulmonary regurgitant fraction (PRF) in patients with a history of repaired tetralogy of Fallot (rTOF) and guides decisions about pulmonary valve replacement (PVR). However, newer techniques such as 3D time-resolved phase contrast (4D flow) MRI are gaining momentum due to the added clinical value beyond conventional flow metrics. While agreement between 2D PC CMR and 4D flow is well-established, the real-world implications for clinical decision-making have not been fully studied and are thus the primary aim of this study. All patients with rTOF who underwent standard CMR plus retrospectively gated 4D flow between February 2021 – June 2023 were identified. Clinical information was collected from the electronic medical record (EMR). 2D cine steady-state free procession (SSFP), PC data, and 4D flow imaging data were analyzed using standard post-processing analysis. Clinical decisions (“intervention vs. no intervention”) were determined using a standardized algorithm, and inter-rater agreement was assessed using the intra-class coefficient (ICC) and the $\:\kappa\:$ coefficient. 33 patients were included. PRF correlated strongly between 2D PC and 4D flow (*r* = 0.87, r_s_ = 0.89) and between 4D flow and volumetric SSFP analysis (*r* = 0.81, r_s_ = 0.79). The mean absolute difference between 2D and 4D PRF was − 3.76% (95% CI [-6.91, -0.60]) with an ICC of 0.83. Inter-rater agreement for the decision was moderate ($\:\kappa\:$=0.58) using only PRF calculated only using 2D PC and 4D flow, and was strong ($\:\kappa\:$=0.76) when using PRF calculated by 2D PC and 4D flow with volumetric PRF. Though clinical indications for PVR in rTOF vary, 4D flow generates accurate measurements of PRF and yields similar clinical decisions about intervention in rTOF. Future studies exploring ventricular volume assessment by 4D flow could potentially eliminate the need for cine SSFP acquisitions and lead to a shorter, more comprehensive CMR exam for TOF patients.

## Introduction

Tetralogy of Fallot (TOF) is the most common cyanotic congenital heart defect (CHD) with an estimated prevalence of 4.6/10,000 new cases per year [1–3]. While surgical outcomes and long-term prognosis have improved significantly, residual anatomic and hemodynamic abnormalities are common [3–6]. Chronic pulmonary regurgitation (PR) is a common hemodynamic complication following initial repair of TOF and frequently leads to right ventricular (RV) dilation and dysfunction with increased risk for exercise intolerance, right heart failure, arrhythmias, and sudden death [3, 7–10] Pulmonary valve replacement (PVR) reduces PR, decreases RV overload, and may normalize RV volumes [4, 11]. However, the hemodynamic benefits of PVR must be balanced with the risks of premature intervention and surgical complications including valve failure and death [4]. These considerations are influenced by growing transcatheter PVR options such that optimal timing of PVR remains highly debated. The decision to pursue intervention involves careful consideration of natural history and pathophysiology as well as procedural risks and benefits [4, 7, 11, 12].

2D phase-contrast (PC) cardiac magnetic resonance (CMR) is currently the gold standard for monitoring PR and pulmonary blood flow in patients with repaired TOF (rTOF) [4, 13, 14]. However, long scan times (often > 60 min) and breath-holding requirements present challenges to clinical workflow and widespread usage, especially in younger patients and those with developmental delay [10, 13, 15]. Additionally, 2D PC CMR may inaccurately quantify flow due to motion of the heart relative to the specified plane and therefore yield a technically limited assessment of complex flow patterns [16]. 3D time-resolved phase contrast (4D flow) CMR is a novel technique that allows magnitude and PC data to be obtained from a single, free-breathing sequence [13, 17]. 4D flow scan time is an average of 5–15 min and thus may decrease the need for anesthesia in younger patients as well as patients with decompensated heart failure or developmental delay, making it a promising new technique for long-term monitoring of rTOF patients [5, 13, 17].

Several studies have shown that 4D flow performs similarly to 2D PC in measurements of blood flow and ventricular volume [13, 18, 19]. In one study of rTOF patients, 4D flow demonstrated greater accuracy compared to 2D PC for flow measurements and performed similarly to 2D steady-state free procession (SSFP) for volumetric analysis [13]. Moreover, the growing availability of commercial software applications has made 4D flow post-processing easier, less time consuming and more clinically feasible. Despite this, adoption into clinical workflow has not become universal. This is likely due to the fact that while agreement between 2D and 4D flow is established, the implications of 4D flow on real-world PVR decision making have not been fully studied [13]. Based on prior data, 4D flow is expected to yield similar decisions about PVR; however, to our knowledge, there have been no studies demonstrating 4D flow’s performance in guiding decisions in true clinical settings. This study aims to retrospectively compare clinical decision making in the management of rTOF using 2D PC CMR versus 4D flow to test the hypothesis that 4D flow provides accurate clinical information that yields similar clinical decisions.

## Materials and methods

### Study Population

We retrospectively identified all patients with rTOF who underwent standard clinical CMR with cine SSFP and PC imaging between February 2021 and June 2023 at Lurie Children’s Hospital. The medical center IRB approved this study, and informed consent was waived for retrospective clinical data collection. Patients who had undergone prior PVR were included. Patients who did not have diagnostic quality CMR exams, who had important metallic artifact, and who did not have both 2D phase contrast and 4D flow imaging were excluded. Demographic data and clinical history were manually extracted from the electronic medical record (EMR) and included patient age, sex, body surface area (BSA), cardiac diagnoses and number of prior cardiac intervention(s), symptoms, and EKG results including QRS duration.

### Image Acquisition

Imaging was performed on a 1.5T scanner (MAGNETOM Aera; Siemens Healthineers, Erlangen, Germany) using an 18-channel matrix coil. Standard balanced SSFP cine imaging and 2D PC scans were obtained per routine clinical protocol and as appropriate for the assessment of patients with TOF [20]. If contrast was administered, 2D PC MRI and 4D flow were acquired following administration of either ferumoxytol (Feraheme, AMAG Pharmaceuticals, Waltham, MA) or gadobutrol (Gadavist, Bayer HealthCare, Whippany, NJ). 4D flow scan characteristics for this cohort were: spatial resolution = 1.4–4.5 × 1.1–3.1 × 1.4–3.5 mm^3^, temporal resolution = 36.0–44.8 ms, repetition time/echo time [TR/TE] = 4.7–5.1/2.2–3.0 ms, flip angle = 15°, and velocity sensitivity (VENC) = 100–250 cm/s. General anesthesia was administered by a pediatric anesthesiologist per the institutional clinical protocol for 9/33 patients (27%). When patient cooperation allowed for breathholding, 2D flow was obtained with breatholds per the clinical standard. Similarly, in the nine scans performed under general anesthesia, breatholding was simulated by holding mechanical ventilation.

### Image Analysis

**Fig. 1:**
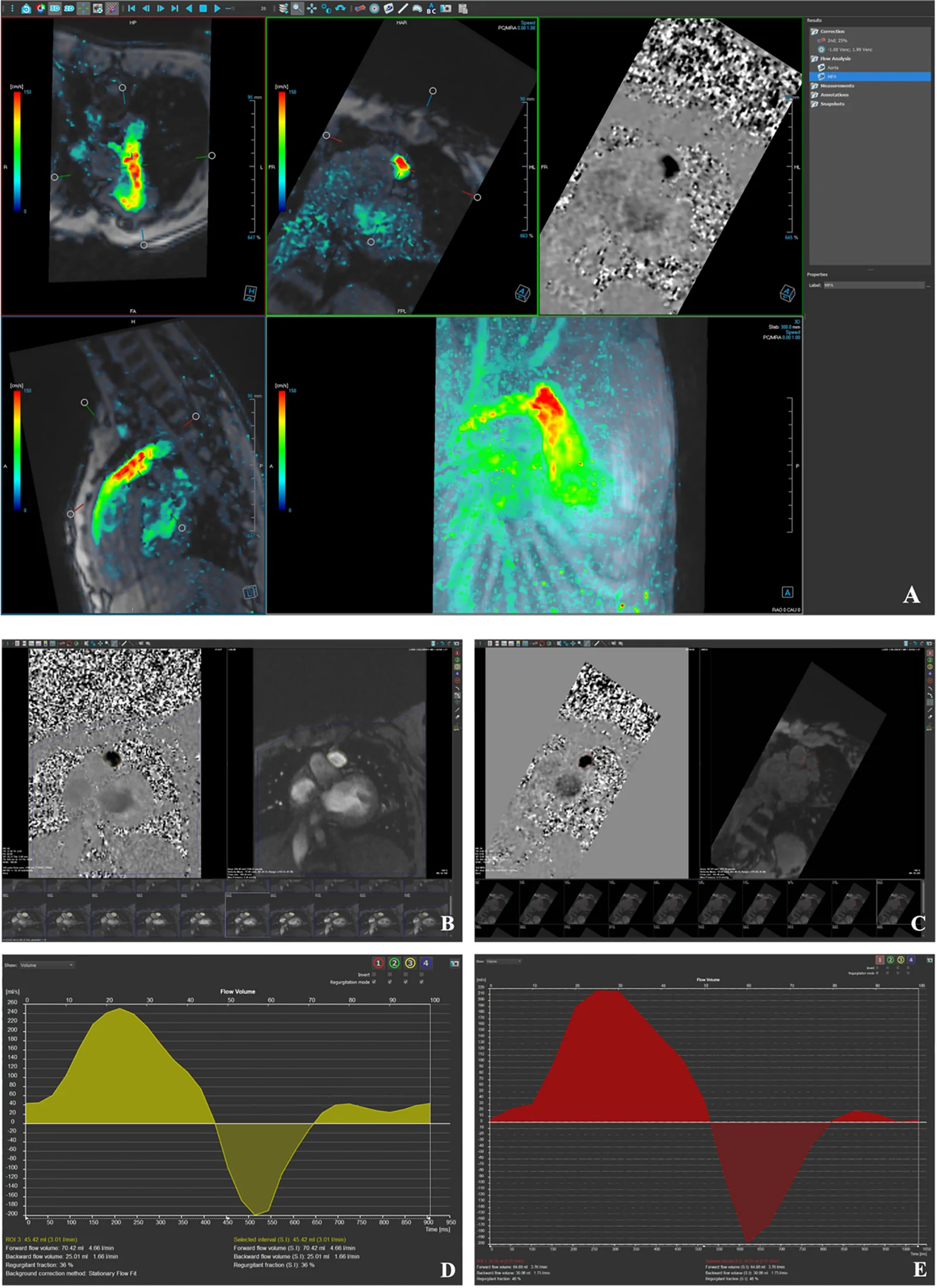
**A** Example of 4D flow visualization and quantification with pulmonary artery plane placement. Region of interest (ROI) placement for 2D PC (**B**) and 4D flow (**C**). Flow-time curves of pulmonary regurgitation using 2D PC (**D**) and 4D flow (**E**)

2D SSFP and PC data were obtained from the clinical CMR report completed by the cardiologist and radiologist. PRF based on volumetric data (PRF_SSFP_) was calculated using the ratio of the stroke volume (SV) difference and the RV stroke volume, as demonstrated by the following equation: PRF_SSFP_ = (SV_RV_ – SV_LV_)/SV_RV_. Medis Suite software (Medis Medical Imaging, Leiden) was used for volumetric analysis and 2D PC and 4D flow post-processing (Fig. 1). Background phase offset corrections were performed, and the phase unwrap tool was applied if there was velocity aliasing For 4D flow analysis, cross-sectional planes were oriented perpendicular to the direction of flow and vessel anatomy. Pulmonary flow was measured at the main pulmonary artery, and the 3D region of interest was manually drawn around the vessel lumen directly above the pulmonary valve. Flow parameters such as net flow, forward flow, backward flow, and PRF were quantified and recorded.

### PVR Decision-Making Algorithm

**Fig. 2:**
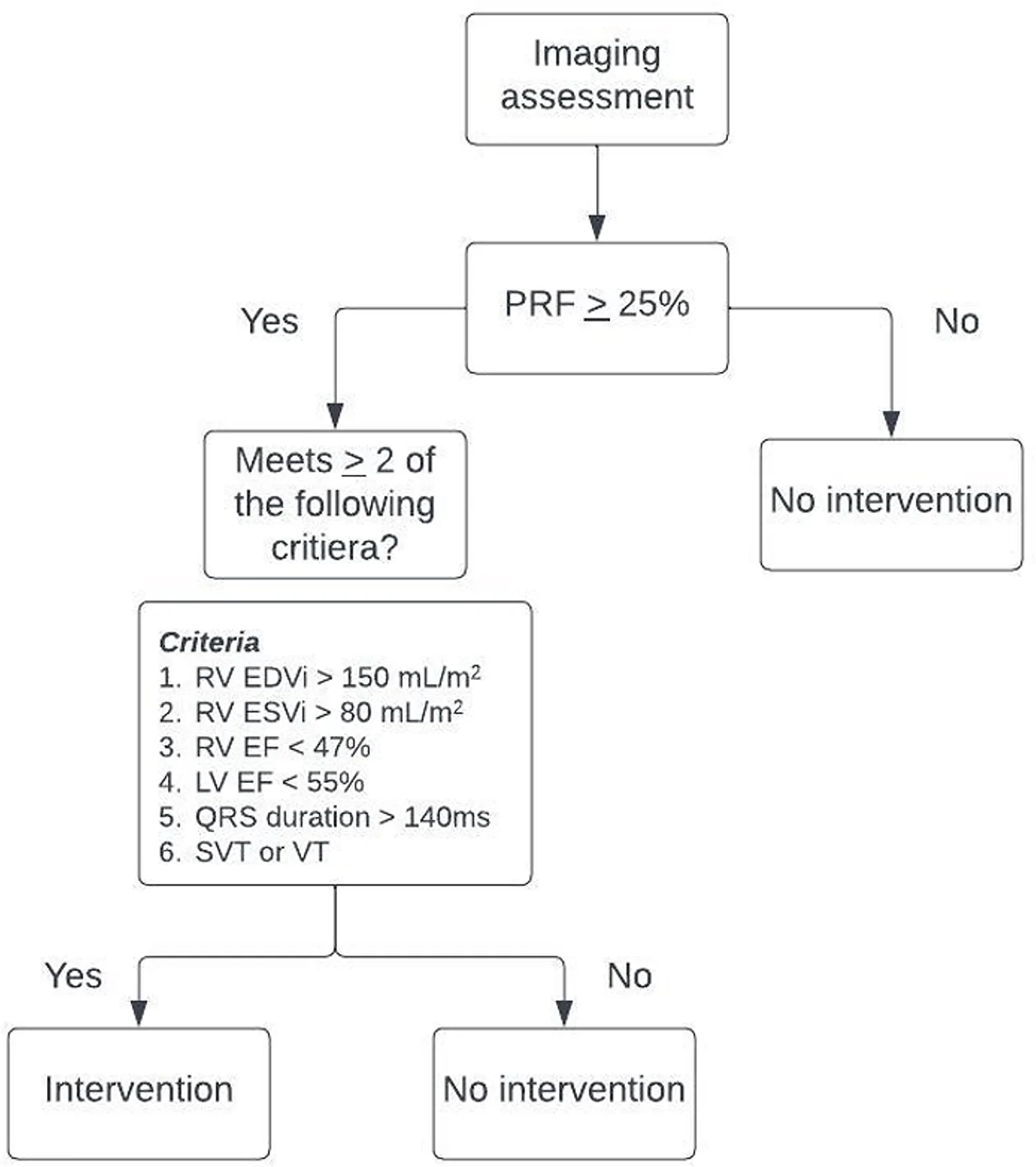
Clinical decision-making algorithm adapted from proposed recommendations based on the current literature [4]

Clinical decisions regarding PVR (“intervention vs. no intervention”) were determined exclusively using the algorithm outlined in the flow chart (Fig. 2) [4]. Clinicians were not involved nor did they influence any decisions. Decisions were first made using the PRF calculated only by 2D PC or 4D flow. The same algorithm was then run again using PRF calculated by 2D PC or 4D flow in conjunction with PRF_SSFP_, similar to conventional clinical practice. In this calculation, the higher of the two values was used for the final determination of PRF. For example, if PRF calculated using 2D/4D PC imaging exceeded 25% whereas the PRF derived from volumetric assessment was below this threshold, the value above 25% would be used in the final decision-making algorithm.

### Statistical Analysis

All continuous variables were reported as mean *±* standard deviation, and categorical variables were reported as percentage and frequency. ANOVA and chi-square tests were utilized to analyze quantitative and categorial demographic data, respectively. Bland-Altman analyses were used to assess agreement between 2D PC and 4D flow. Inter-rater agreement for the decision was measured using the intra-class coefficient and Cohen’s kappa coefficient. The kappa statistic was interpreted in accordance with the Landis and Koch (1977) scale: 0.2–0.4 represents fair agreement, 0.41–0.6 represents moderate agreement, 0.61–0.8 represents substantial agreement, and greater than 0.8 represents near perfect agreement [21].

## Results

**Fig. 3:**
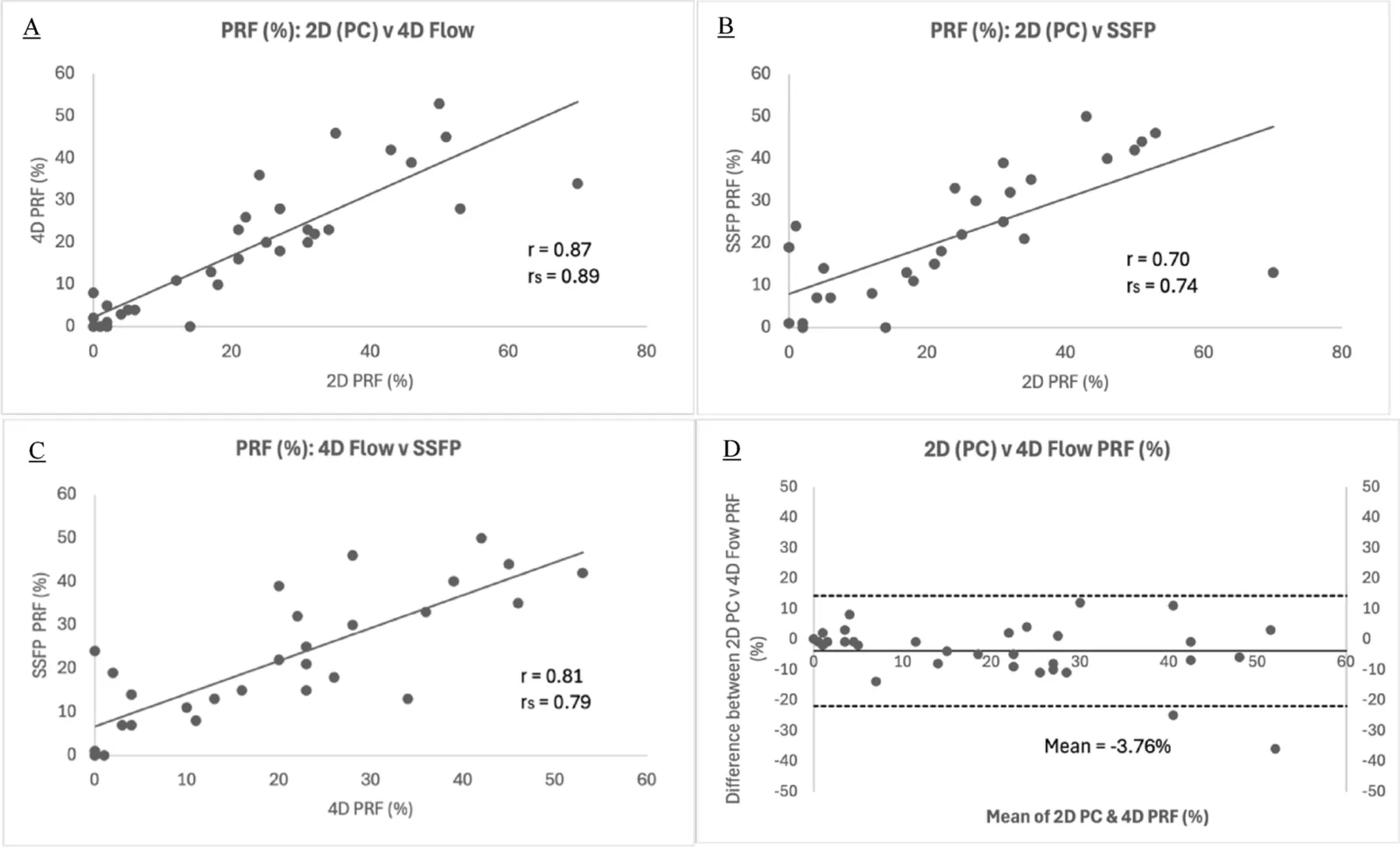
Scatter plots demonstrating correlation between different measurements of pulmonary regurgitant fraction (PRF). **A** PRF_2D_ and PRF_4D Flow_ **B** PRF_2D PC_ and PRF_SSFP_ **C** PRF_4D Flow_ and PRF_SSFP_ **D** Bland-Altman plot comparing PRF calculated by 2D PC and 4D flow. Pearson correlation coefficients (r) and Spearman correlation coefficients (r_s_) are displayed on each correlation graph. On the Bland Altmann plots, the upper and lower limits of agreement are 14.40 and − 21.89, respectively. The mean difference between 4D flow-derived PRF and 2D PC-derived PRF was − 3.76% (95% CI [−6.91, −0.60]) with an intra-class coefficient of 0.83

Fifty patients met initial criteria during the defined study time period. Fifteen patients were excluded due to lack of 2D (*n* = 6) or 4D (*n* = 9) data, and two patients were excluded due to metallic artifact precluding PV assessment, resulting in a study population of 33 patients. Contrast was administered in 15/33 exams (45%). Patient demographics are shown in Table 1. Important differences among groups stratified by clinical decision include BSA-indexed RV end-diastolic volume (EDVi), BSA-indexed RV end-systolic volume (ESVi), LV ejection fraction (EF), and PRF measured by 2D PC and 4D flow. PRF calculated by 2D PC correlated strongly with 4D flow-derived PRF (*r* = 0.87, r_s_ = 0.89) (Fig. 3). PRF showed moderate-strong correlation between 2D PC and SSFP (*r* = 0.70, r_s_ = 0.74) and between 4D flow and SSFP (*r* = 0.81, r_s_ = 0.79) (Fig. 3). The mean difference between 4D flow-derived PRF and 2D PC-derived PRF was − 3.76% (95% CI [−6.91, −0.60]) with an intra-class coefficient of 0.83 (Fig. 3).

Algorithm-based decisions for PVR are shown in Table 2. 28 decisions (85%) were concordant between 2D PC and 4D flow. Intervention was recommended in 23 of these cases, and no intervention was recommended in the other 5. There were five discordant decisions; in all five, 2D recommended intervention whereas 4D recommended no intervention. When using PRF calculated by 2D only and 4D only, the inter-rater agreement was moderate (*κ* = 0.58). When incorporating PRF_SSFP_ into the decision, there were three discordant decisions, in which 2D recommended intervention whereas 4D recommended no intervention. The inter-rater agreement improved and was strong (*κ* = 0.76).

**Table 1:** Demographic, clinical, and radiographic information of repaired tetralogy of Fallot patients stratified based on clinical decision regarding pulmonary valve replacement (PVR)

	Overall Patient Demographics	Patient Demographics by Clinical Decision			
	Total (*n* = 33)	No Intervention (*n* = 23)	Disagreement (*n* = 5)	Intervention (*n* = 5)	*p* value
***Demographics***					
Age (years)	19.2 ± 12.4	19.9 ± 13.4	24.1 ± 8.2	10.7 ± 7.0	0.21
Sex (% male)	57.6% (*n*=19)	47.8% (*n*=11)	60.0% (*n*=3)	100.0% (*n*=5)	0.10
Weight (kg)	56.6 ± 28.6	57.2 ± 26.3	71.9 ± 34.4	38.8 ± 29.4	0.18
Height (cm)	149.9 ± 24.2	149.1 ± 21.8	164.1 ± 10.2	139.1 ± 39.5	0.26
BSA (m^2^)	1.5 ± 0.5	1.5 ± 0.4	1.8 ± 0.5	1.2 ± 0.6	0.16
% pulm atresia	21.2 (*n*=7)	17.4% (*n*=4)	20.0% (*n*=1)	40.0% (*n*=2)	0.53
Primary or staged repair:					0.24
Primary	54.5% (*n*=18)	60.9 (*n*=14)	20.0% (*n*=1)	60.0% (*n*=3)	–
Staged	45.5% (*n*=15)	39.1% (*n*=9)	80.0% (*n*=4)	40.0% (*n*=2)	–
Repair type:					0.30
Transannular patch	45.5% (*n*=15)	39.1% (*n*=9)	60.0% (*n*=3)	60.0% (*n*=3)	–
Valve-sparing	27.3% (*n*=9)	30.4% (*n*=7)	0% (*n*=0)	40.0% (*n*=2)	–
Conduit	21.2% (*n*=7)	26.1% (*n*=6)	20.0% (*n*=1)	0% (*n*=0)	–
Unknown	6.1% (*n*=2)	4.3% (*n*=1)	20.0% (*n*=1)	0% (*n*=0)	–
***Volume/Function***					
RV EDVi (mL/m^2^)	118.7 ± 35.6	109.0 ± 30.1	123.8 ± 16.5	158.2 ± 48.5	0.01*
RV ESVi (mL/m^2^)	62.4 ± 23.8	55.0 ± 20.0	72.0 ± 17.1	86.6 ± 29.1	0.01*
RV SV (mL)	80.0 ± 23.3	78.3 ± 23.3	95.0 ± 23.5	73.2 ± 21.1	0.28
RV EF (%)	48.4 ± 7.2	50.1 ± 7.4	44.0 ± 6.6	45.0 ± 4.8	0.12
LV SV (mL)	66.8 ± 21.1	68.0 ± 20.8	77.8 ± 15.8	50.2 ± 20.5	0.10
LV EF (%)	54.8 ± 6.6	56.7 ± 6.4	51.0 ± 4.1	49.4 ± 5.2	0.02*
PRF (2D PC) (%)	22.0 ± 18.7	15.0 ± 15.7	29.6 ± 3.6	47.0 ± 16.7	0.0003*
PRF (SSFP) (%)	21.6 ± 15.0	17.6 ± 15.1	26.8 ± 8.3	33.2 ± 12.9	0.08
PRF (4D flow) (%)	18.3 ± 15.7	13.5 ± 14.9	20.8 ± 2.2	37.8 ± 11.2	0.003*

P values assessing for statistical significance between clinical outcomes were calculated using chi square for categorical data (sex, presence of pulmonary atresia, primary or secondary repair, and type of repair) and ANOVA for quantitative data (age, weight, height, BSA, and volume/function data) with * indicating significance (*p* < 0.05). EDVi = indexed end diastolic volume; ESVi = indexed end systolic volume; SV = stroke volume; EF = ejection fraction; PRF = pulmonary regurgitant fraction

**Table 2:** Top pane shows clinical decisions made using the pulmonary regurgitant fraction (PRF) calculated only by 2D PC or 4D flow

		**PRF**_**4D Flow**_		**Inter-rater agreement (K): 0.58**
		No intervention	Intervention	
**PRF**_**2D PC**_	No intervention	23	0	
	Intervention	5	5	
		**PRF**_**4D Flow**_ **+PRF**_**SSFP**_		**Inter-rater agreement (K)**: 0.76
		No intervention	Intervention	
**PRF**_**2D PC**_ **+ PRF**_**SSFP**_	No intervention	23	0	
	Intervention	3	7	

Bottom pane shows decisions made using the same algorithm but with PRF calculated by 2D PC (_PRF2D PC_) or 4D flow (PRF_4D Flow_) in conjunction with volumetric PRF (PRF_SSFP_) calculated using stroke volumes. When using both PRF calculated by 2D or PC and PRF calculated using cine SSFP data, the higher of the two values was used for the final determination of PRF

## Discussion

In this study, we assessed the utility of 4D flow compared to 2D PC in real-world clinical decision-making regarding indication for PVR in the rTOF population. We also compared PRF measured by 4D flow, 2D PC, and SV difference and found strong correlations between PRF measured via all three methods. These findings are consistent with prior studies that similarly found robust agreement between flow measurements obtained via 2D PC and 4D flow [18, 19]. Though this is still a growing area of work, this study adds to the existing literature supporting 4D flow as an accurate and reliable way of obtaining flow measurements for this unique patient population. We also found that 4D flow and 2D PC resulted in similar clinical decisions about recommendation for PVR using a conventional algorithm. Using all available information (both flow and cine SSFP derived) resulted in nearly universal agreement. Based on these findings, 4D flow holds significant promise in the evaluation and management of patients with rTOF.

This study offers novel insight into the role of 4D flow in conventional decision-making for patients with rTOF. Despite several studies investigating ideal indications for intervention, the decision surrounding intervention remains highly nuanced and individualized with significant practice variation across centers and providers [22, 23]. Several studies have found that intervention in patients satisfying volumetric requirements leads to improvement in RV volume and function, yet the specific thresholds predictive of optimal outcome vary across studies [24–28]. Massarella et al. examined referral patterns in a multicenter cohort of rTOF patients and found that majority of their cohort met criteria for PVR yet were not referred for intervention [22]. Further analysis of their cohort suggested that thresholds for referral proposed in guidelines are more aggressive than the thresholds used to guide decision-making in everyday clinical practice [22]. The small differences in agreement across clinical decisions found in our study therefore fall well within the range of practice variation. Additionally, this hightlights the need for further clarification and the potential development of more standardized, evidence-based referral criteria - an area where 4D shows considerable promise and may offer significant contributions.

In addition to 4D flow’s strong clinical performance in determining the severity of PR and indication for PVR, this technique offers additional insights into higher order fluid dynamics and evolving hemodynamic efficiencies [5]. Physiological parameters derived from 4D flow like kinetic energy (KE), helix and vortex formation, energy loss, and wall shear stress have shown promise as early biomarkers in TOF [29–37]. RV KE has been shown to be higher in TOF patients compared to healthy controls and correlate with volumetric and flow markers of disease severity, including RV EDVi and PRF [29, 32–34]. Altered hemodynamic forces and flow patterns, including pathologic vortex formation, are seen in TOF patients and have been shown to correlate with RV dysfunction and exercise intolerance [30, 35–37]. These higher order hemodynamics are promising biomarkers that, with further research and validation, may help establish new criteria for PVR referral and refine current indications for referral.

Limitations of this study include our small single-center cohort of patients with rTOF and retrospective study design, which may limit the applicability of these findings to other populations. Another limitation of this study is the focus on agreement between 2D PC and 4D flow; future study of ventricular volume assessment by 4D flow or in combination with other accelerated techniques could lead to an even shorter, comprehensive exam for TOF patients.

In summary, 4D flow correlates with well-established 2D techniques and has demonstrated a robust ability to reliably and accurately quantify flow parameters in patients with rTOF. The ability to capture conventional and novel hemodynamic parameters in under ten minutes with a single, free-breathing, scan combined with the recent advancements in commercially available post-processing software makes 4D flow an increasingly feasible and compelling addition to the current clinical workflow for management of patients with rTOF [38].

## Data Availability

No datasets were generated or analysed during the current study.

## Abbreviations

CHD: Congenital heart disease
CMR: Cardiac magnetic resonance
LV: Left ventricle
RV: Right ventricle
SV: Stroke volume
TOF: Tetralogy of Fallot
PC: Phase contrast
PR: Pulmonary regurgitation
PRF: Pulmonary regurgitant fraction
PVR: Pulmonary valve replacement
rTOF: Repaired tetralogy of Fallot
